# Role of Comprehensive Renal Genetic Testing in Diagnosing a *RMND‐1* Mitochondrial Disease in Two Adult Cases Exhibiting Variable Disease Phenotypes

**DOI:** 10.1002/ccr3.70421

**Published:** 2025-04-15

**Authors:** Quinn Stein, Ryan Mascarenhas, Sumit Punj, Maggie Westemeyer, Emily Hendricks, Tessa Pitman, Meg Hager, Nour Al Haj Baddar, Kristen Connors, Alexa Bacher, Robin Larson, Lauren Zec, Jennifer Stoddard

**Affiliations:** ^1^ Natera Inc. Austin Texas USA; ^2^ Colorado Kidney Care Lone Tree Colorado USA; ^3^ Boston University Boston Massachusetts USA; ^4^ Pinehurst Nephrology Associates Pinehurst North Carolina USA

**Keywords:** genetic testing, kidney gene panel, *RMND1*, *RMND1*‐related mitochondrial disease

## Abstract

*RMND1‐*related mitochondrial disease is a rare genetic condition that affects multiple organs, including the kidneys. We describe two adult patients whose diagnosis, initiated in childhood, was established through renal gene panel testing, emphasizing the value of genetic testing in uncovering kidney‐related conditions that have a high degree of clinical heterogeneity.

## 1 Introduction

Chronic kidney disease (CKD) refers to a group of complex conditions that, together, impact more than 10% of the global population [1]. CKD becomes more common with increasing age and is most common in the elderly [2]. Globally, CKD is most commonly attributed to diabetes and/or hypertension, but genetic factors are also common [3]. Monogenic causes of CKD are becoming increasingly appreciated, with recent data indicating that genetic causes can be identified in approximately 20% of adults and up to 70% of pediatric patients [4, 5, 6, 7]. Among genetic CKD disorders, autosomal dominant polycystic kidney disease (ADPKD) is the most prevalent, affecting about 1 in every 400–1000 individuals [8]. Rare genetic kidney diseases can be especially challenging to diagnose and treat due to their low prevalence and complex presentation. *RMND1*‐related disease is one such rare condition, often involving kidney dysfunction alongside multisystem manifestations due to its role in mitochondrial protein synthesis.

*RMND1* (Required for Meiotic Nuclear Division Protein 1) is a nuclear‐encoded gene whose protein belongs to the conserved sif2 family of proteins [9]. The RMND1 protein is thought to be localized in the mitochondrial inner membrane complex and plays an integral role in the translation of mtDNA‐encoded polypeptides and oxidative phosphorylation [10, 11].

Biallelic pathogenic variants in *RMND1* are historically associated with the autosomal recessive mitochondrial condition, combined oxidative phosphorylation deficiency‐11 [9], one of several dozen subtypes of combined oxidative phosphorylation deficiency [12]. Clinically, this gene‐disease association (GDA) has been reported as an infantile‐onset multisystem disease characterized by kidney dysfunction, severe hypotonia, white matter encephalopathy, sensorineural hearing loss, global developmental delay, short stature, premature ovarian failure, and lactic acidosis [9, 10, 13]. More recently, case reports have described patients presenting with a more varied and, in some cases, milder phenotype; these reports have proposed new condition names such as “*RMND1*‐related mitochondrial disease” [13, 14] and “Perrault‐like syndrome” [15, 16, 17] to underscore the spectrum of findings. Here we use the nomenclature *RMND1*‐related mitochondrial disease to describe this ultra‐rare genetic condition with less than 100 cases reported in the literature thus far [18]. Patients with *RMND1*‐related mitochondrial disease have a significantly higher likelihood of developing kidney disease, potentially due to the enrichment of mitochondria in renal tubular cells (due to high ATP demands) [14, 19]. *RMND1*‐related kidney disease can include tubulopathies, renal tubular acidosis, interstitial nephritis and may progress to end‐stage kidney disease (ESKD) [14].

The most common pathogenic *RMND1* variant, [NM_017909.4] c.713A > G [18], has been described in both the compound heterozygous and homozygous states, most often in children. To date, the homozygous presence of this variant has been reported in eight children (ages 0–17 years) [13, 14, 15, 16, 18, 20] and two adults (ages 25 and 61 years) [21, 22]. In this report, we describe two additional adults with homozygous *RMND1* c.713A > G variants exhibiting a broad spectrum of disease presentations, thus expanding the reported disease phenotype. These patients' diagnostic journeys began in childhood and were resolved in adulthood using a 385‐gene panel test for common and rare genetic kidney diseases (the Renasight test, Natera Inc.) [4].

## 2 Case History/Examination

### 2.1 Case 1

A 29‐year‐old female patient presented at age 3 with spontaneous pneumothorax and sensorineural hearing loss. The patient had nocturnal enuresis until age 12 and was diagnosed with hypergonadotropic amenorrhea, ovarian dysgenesis, and hypertension as a teenager. She was prescribed oral contraceptives, leading to breast development, and menarche began at age 18. The patient was noted for the absence of neurological problems, attended college, and grew to 6′1″ tall. She uses hearing aids and communicates through verbal speech and lip reading. At age 22, the patient was diagnosed with chronic kidney disease (CKD) stage 3A, which progressed to CKD stage 4 at age 29. Her CKD reversed to CKD 3B as a result of blood pressure control and lifestyle changes. The patient's urine sediment was bland.

### 2.2 Case 2

A 42‐year‐old female presented at age 2 with progressive, profound hearing loss and strabismus. At age 12, the patient was < 5th percentile for height and weight and was diagnosed with both ovarian dysfunction and growth hormone deficiency. As a teenager, the patient was given growth hormone treatment and reached a final height of 5′1″. The pediatric endocrinology evaluation noted short neck, low‐set ears, low posterior hairline, and minimal cubitus valgus. Karyotyping revealed a normal chromosome complement, which ruled out Turner syndrome. Given the overall clinical presentation, multiple pediatric syndromes were considered, but none could account for all the symptoms. As a teenager, she developed a seizure disorder, migraines, tertiary hyperparathyroidism with hypercalcemia, and at age 19, underwent subtotal parathyroidectomy. The patient uses sign language for communication.

The patient experienced kidney failure at age 12. Renal pathology demonstrated multiple fluid‐filled cysts. The interstitium was characterized by chronic inflammation of the medullary compartment with fibroplasia. The cortex was marked by large areas of cortical nephron loss with focal segmental sclerosis of the remaining glomeruli. Tubules demonstrated thyroidization and cyst formation. The patient received a kidney transplant from her mother at age 12 and has continued to have a normal kidney graft function, with no recurrence of CKD, through the most recent follow‐up.

Currently, the patient lives with her parents and remains dependent for living and care assistance, managing her medical visits and medication, and other complex tasks. The patient is independent in her general self‐care and is capable of working outside the home in certain capacities.

## 3 Methods (Differential Diagnosis)

These patients' diagnostic journeys began in childhood and were resolved in adulthood using a 385‐gene panel test for common and rare genetic kidney diseases (the Renasight test, Natera Inc.).

## 4 Conclusions and Results (Outcome and Follow‐Up)

### 4.1 Case 1

Genetic testing at age 29 identified the homozygous *RMND1* (c.713A > G) variant. No other clinically relevant variants were detected. This precipitated the patient's first diagnosis, at age 29, with *RMND1*‐related mitochondrial disease.

### 4.2 Case 2

Genetic testing at age 42 identified homozygous *RMND1* (c.713A > G) variant, which are thought to explain her clinical findings. No other clinically relevant variants were detected. This precipitated the patient's first diagnosis, at age 42, with *RMND1*‐related mitochondrial disease.

## 5 Discussion

Very few individuals with *RMND1*‐related conditions have been described, with children and adolescents with homozygous c.713A > G variants comprising the majority of those reported [13, 14, 15, 16, 18]. Only two adults with *RMND1*‐related conditions have been described to date, one male and one female with variable phenotypes (Table 1) [21, 22]. Our report adds two additional adult female cases to the literature, expanding the current knowledge of the wide phenotypic spectrum associated with this condition. Several points can be made from the two cases described in this report: (1) Adults with *RMND1‐*related conditions can display extreme variable expressivity; (2) kidney transplant can be an effective long‐term treatment for the renal aspects of this condition; (3) a clinician ordering a comprehensive kidney gene panel that contains many genes has a greater likelihood of detecting rare and ultra‐rare conditions such as *RMND1*‐related disorders; and (4) including genes associated with rare and ultra‐rare conditions on gene panels can aid in disease identification in patients who have gone years without a diagnosis.

**TABLE 1:** A comparison of the symptoms and characteristics associated with *RMND1*‐related mitochondrial disease between previously reported adult cases and the two adult cases in this report.

Characteristic	Kömhoff [21]	Rioux [22]	Patient 1	Patient 2
Sex	Male	Female	Female	Female
Age at last report (Y, years)	25	61	29	42
ID, DD, FTT	Yes, presented at 6 weeks of age with FTT and vomiting	No	No	Yes
Sensorineural hearing loss	Yes	Yes	Yes	Yes
Vision loss	NR	NR	No	Strabismus, surgical intervention at age 2
Ovarian dysfunction	Not applicable	Hypergonadotropic amenorrhea, streak ovaries, began OCPs	Ovarian dysfunction diagnosed at young age	Ovarian sufficiency and delay in sexual maturation
Musculoskeletal	Lower limb spasticity	Elevated serum CK, osteoporosis	Tall stature (6′1″)	Required growth hormone to reach 5′1″
Renal impairment	Severely decreased renal function, CKD4 at age 24	Mild kidney atrophy at age 24 with steady decline, renal failure at age 61	CKD3A at age 22 progressed to CKD4 at age 29 before reversing to CKD3b	Renal failure at age 12
Kidney transplant	Yes, at age 25	No (pre‐transplant evaluation)	No	Yes, at age 12
Other	Hyporeninemic hypoaldosteronism	Mild leukopenia, skin cancer between ages 55 and 61.	Spontaneous pneumothorax at age 3. Enuresis until age 12.	NR

Abbreviations: CK, creatine kinase; CKD, chronic kidney disease; DD, developmental disability; FTT, failure to thrive; ID, intellectual disability; Ktx, kidney transplant; NR, not reported; OCP, oral contraceptives.

The two adult female cases described in this report demonstrated remarkable variable expressivity of the condition despite having the same homozygous *RMND1* variant. Marked differences between presentations in both cases included findings related to stature and intellectual abilities. Patient 1 reached an adult height of 6′1″, unaided by treatment, whereas patient 2 required growth hormone therapy to achieve an adult height of 5′1″. Additionally, patient 2 required special education for an intellectual disability and lives with her family for assistance, while patient 1 is a college graduate and recently married. Similarities in disease symptoms between the cases included findings related to reproductive and sensorineural health like hearing loss and ovarian dysfunction, which have emerged as part of the *RMND1* phenotype [15, 16, 22].

While kidney involvement appears to be relatively uncommon within mitochondrial diseases as a group, affected patients with *RMND1*‐related mitochondrial disease have a significantly higher likelihood of developing kidney disease [14, 18, 23]. The variability and risk of kidney disease progression remain unclear. Patient 1 currently has CKD3b and appears stable, while Patient 2 underwent a living‐related kidney transplant from her mother within 4 months of initiating dialysis at age 12. The two previously reported adult cases with *RMND1*‐related mitochondrial disease with the same *RMND1* variant as these two patients had kidney function impairment that progressed to kidney failure, one of whom also had a kidney transplant at age 25 (Table 1) [21, 22]. The range of kidney function impairment among these four patients demonstrates a consistent but variable kidney involvement in the *RMND1*‐related mitochondrial disease progression.

We demonstrate that kidney transplant intervention can achieve long‐term success, as shown by Patient 2, whose allograft continues to function well 31 years post‐transplant. In the literature, a total of eight cases with *RMND1*‐related mitochondrial disease underwent kidney transplantation. The two previously mentioned adult cases with the same *RMND1* variant as the patients reported here received kidney transplants at ages 25 [21], (Table 1) and 9, respectively [13]. No further follow‐up information was available for either case beyond those ages. The remaining six cases had different *RMND1* variants and all received kidney transplants before age 18, also with limited follow‐up information [13, 14, 24]. Among these cases, the longest documented transplant success was 6 years, based on available follow‐up data, as reported in 2016 [24]. As such, patient 2, reported here, is the longest documented surviving kidney transplant case with *RMND1*‐related mitochondrial disease.

When evaluating genes to be included on a panel, multiple aspects of the gene‐disease association should be considered. The inclusion of only genes associated with highly prevalent diseases could result in missing the identification of rare genetic conditions in affected patients. Use of a next generation sequencing (NGS)‐based gene panel allows querying of a range of genes, including both common and rare genetic conditions. In the cases presented here, it was both the decision of the genetic testing lab to include *RMND1* on an NGS‐based panel and the nephrologist's decision to utilize a comprehensive kidney gene panel that ultimately led to the diagnosis of these two patients with *RMND1*‐related mitochondrial disease. This demonstrates that the use of a comprehensive kidney gene panel can increase the likelihood of identifying the accurate diagnosis.

A kidney gene panel is often the most practical and efficient diagnostic tool for nephrologists, as it focuses specifically on genetic conditions related to kidney disease—conditions well within the nephrologist's scope of practice. It allows for a targeted approach to diagnosis, making it more accessible and streamlined for clinicians who specialize in kidney‐related issues. While whole exome and whole genome sequencing can also provide the same results and even offer a broader diagnostic scope, including the potential to detect additional, rare conditions and incidental findings, they tend to be more complex and may not be as readily accessible or familiar to nephrologists. Exome and genome sequencing and rapid genome sequencing have become increasingly integral in diagnosing patients with multiple congenital anomalies and are typically used in cases where a patient presents with a broader range of symptoms that span multiple organ systems, such as in critically ill newborns or children with a variety of health conditions [25]. For patients with kidney disease, however, a kidney gene panel is generally sufficient and ensures a more focused, efficient investigation. Ultimately, the choice of diagnostic approach depends on the specific case and clinical context.

Here, genetic testing provided answers to patients and their healthcare teams who have gone decades without a correct diagnosis [26, 27, 28]. For many years, symptoms in patient 1 were all thought to be unrelated. Patient 2 was given a working diagnosis of CHARGE syndrome (coloboma, heart disease, atresia of the choanae, retarded growth and mental development, genital anomalies, and ear malformations and hearing loss) as a child. The decades‐long diagnostic odyssey resolved for both patients in adulthood after genetic testing was ordered through nephrology. Benefits to receiving a diagnosis include medical, emotional, psychological, cognitive, and financial advantages [6, 27]. In these cases, the diagnosis provided patients and their families with the relief of finally having a correct diagnosis, a defined name of the condition, answers to previously unresolved questions, and a community for support. These cases exemplify the recent recommendations from the National Kidney Foundation to perform genetic testing for symptomatic adult or pediatric patients presenting with multi‐organ syndromes of unknown etiology, CKD or ESKD of unknown origin, or when genetic testing could potentially end a diagnostic odyssey [6].

With an increase in utilization of exome and genome sequencing technologies, especially in the neonatal period [29], earlier diagnoses of *RMND1*‐related mitochondrial disease may occur in the future. Initial medical management recommendations for *RMND1*‐related mitochondrial disease should include referral to biochemical medical genetics for evaluation and coordination of care given the breadth and variable expressivity of symptoms related to this condition [18]. Evaluations for a newly diagnosed patient should include: Audiology for sensorineural hearing loss; Endocrinology for failure to thrive, growth retardation, and parathyroid abnormalities; Cardiology for congenital heart defects, arrhythmias, and hypertension; Nephrology for kidney dysfunction; Neurology for hypotonia, structural neurological abnormalities, and seizures; Ophthalmology for visual abnormalities; formal developmental evaluation, and consideration of Reproductive Endocrinology for discussion of fertility preservation (as is sometimes done for Turner syndrome) [18, 22, 30].

Our report demonstrates the value of pursuing comprehensive genetic testing in uncovering the diagnosis of an ultra‐rare kidney‐related conditions that has a high degree of clinical heterogeneity.

## Author Contributions

**Quinn Stein:** conceptualization, data curation, formal analysis, investigation, methodology, project administration, resources, supervision, visualization, writing – original draft, writing – review and editing. **Ryan Mascarenhas:** conceptualization, data curation, formal analysis, investigation, methodology, resources, writing – original draft, writing – review and editing. **Sumit Punj:** conceptualization, data curation, formal analysis, investigation, methodology, resources, writing – original draft, writing – review and editing. **Maggie Westemeyer:** conceptualization, data curation, formal analysis, investigation, methodology, resources, writing – original draft, writing – review and editing. **Emily Hendricks:** conceptualization, data curation, formal analysis, investigation, methodology, resources, writing – original draft, writing – review and editing. **Tessa Pitman:** conceptualization, data curation, formal analysis, investigation, methodology, resources, writing – original draft, writing – review and editing. **Meg Hager:** conceptualization, data curation, formal analysis, investigation, methodology, resources, writing – original draft, writing – review and editing. **Nour Al Haj Baddar:** conceptualization, data curation, formal analysis, investigation, methodology, resources, writing – original draft, writing – review and editing. **Kristen Connors:** conceptualization, data curation, formal analysis, investigation, methodology, resources, writing – original draft, writing – review and editing. **Alexa Bacher:** conceptualization, data curation, formal analysis, investigation, methodology, resources, writing – original draft, writing – review and editing. **Robin Larson:** conceptualization, data curation, formal analysis, investigation, methodology, resources, writing – original draft, writing – review and editing. **Lauren Zec:** conceptualization, data curation, formal analysis, investigation, methodology, resources, writing – original draft, writing – review and editing. **Jennifer Stoddard:** conceptualization, data curation, formal analysis, investigation, methodology, resources, writing – original draft, writing – review and editing.

## Ethics Statement

The authors have nothing to report.

## Consent

Written informed consent for publication was obtained from the patients and their families described in this case report.

## Conflicts of Interest

R. Mascarenhas and K. Connors declare no conflicts of interest. J. Stoddard is a consultant for Medtronic and a speaker for Boehringer‐Ingelheim. The remaining authors are employees of Natera Inc. and hold stocks or stock options in the company.

## Data Availability

The data generated and/or analyzed during this study are not publicly available due to privacy and ethical restrictions but can be obtained from the corresponding author upon reasonable request.
